# Multisite Evaluation of Cepheid Xpert Carba-R Assay for Detection of Carbapenemase-Producing Organisms in Rectal Swabs

**DOI:** 10.1128/JCM.00341-16

**Published:** 2016-06-24

**Authors:** M. Tato, P. Ruiz-Garbajosa, M. Traczewski, A. Dodgson, A. McEwan, R. Humphries, J. Hindler, J. Veltman, H. Wang, R. Cantón

**Affiliations:** a Servicio de Microbiología, Hospital Ramón y Cajal and Instituto Ramón y Cajal de Investigación Sanitaria (IRYCIS), Madrid, Spain; b Clinical Microbiology Institute, Wilsonville, Oregon, USA; c Central Manchester University Hospitals NHS Foundation Trust, Manchester Royal Infirmary, Manchester, United Kingdom; d Department of Pathology & Laboratory Medicine, David Geffen School of Medicine, University of California Los Angeles, Los Angeles, California, USA; e Division of Infectious Diseases, Wayne State University School of Medicine, Detroit, Michigan, USA; f Department of Pathology, New York Medical College and Department of Pathology and Clinical Laboratories, Westchester Medical Center, Valhalla, New York, USA; The Johns Hopkins University School of Medicine

## Abstract

Rapid identification of patients who are colonized with carbapenemase-producing organisms (CPO) is included in multiple national guidelines for containment of these organisms. In a multisite study, we evaluated the performance of the Cepheid Xpert Carba-R assay, a qualitative diagnostic test that was designed for the rapid detection and differentiation of the *bla*_KPC_, *bla*_NDM_, *bla*_VIM_, *bla*_OXA-48_, and *bla*_IMP-1_ genes from rectal swab specimens. A double rectal swab set was collected from 383 patients admitted at four institutions (2 in the United States, 1 in the United Kingdom, 1 in Spain). One swab was used for reference culture (MacConkey broth containing 1 mg/liter of meropenem and subcultured to a MacConkey agar plate with a 10-μg meropenem disk) and for sequencing of DNA obtained from carbapenem-nonsusceptible isolates for carbapenemase identification. The other swab was used for the Xpert Carba-R assay. In addition to the clinical rectal swabs, 250 contrived specimens (108 well-characterized CPO and 142 negative controls spiked onto negative rectal swabs) were tested. Overall, 149/633 (23.5%) samples were positive by the Xpert Carba-R assay. In 6 samples, multiple targets were detected (4 VIM/OXA-48, 1 IMP-1/NDM, and 1 NDM/KPC). The Xpert Carba-R assay detected 155 targets (26 IMP-1, 30 VIM, 27 NDM, 33 KPC, 39 OXA-48) within a time range of 32 to 48 min. The sensitivity, specificity, and positive and negative predictive values of the Xpert Carba-R assay compared to those of the reference culture and sequencing results were 96.6% (95% confidence interval [CI], 92.2% to 98.9%), 98.6% (95% CI, 97.1% to 99.4%), 95.3%, and 99.0%, respectively. The Cepheid Xpert Carba-R assay is an accurate and rapid test to identify rectal colonization with CPO, which can guide infection control programs to limit the spread of these organisms.

## INTRODUCTION

The global spread of carbapenemase-producing organisms (CPO) has been highlighted by international health authorities as a critical public health concern (1–3). Containment of this spread, currently recommended in North American and European infection control guidelines, includes accurate and rapid identification of colonization with CPO (4, 5). Different methods for detection of CPO in rectal swabs have been used, including culture with specific chromogenic media and in-house and commercial molecular tests (6, 7). Culture-based methods are limited by sensitivity and specificity issues depending on the composition of the medium and the targeted carbapenemases (e.g., KPC, VIM, NDM, IMP, or OXA-48 enzymes), the requirement of 24 to 48 h for growth, and the need for confirmation with phenotypic and/or molecular methods. Moreover, as the accuracy of culture methods is highly dependent on medium formulation (type and concentration of antibiotic supplementation), the increasing diversity of carbapenemase enzymes and their variable expression affect the sensitivity of culture methods (8, 9). In addition, bacterial load in rectal or perirectal swabs also impacts the accuracy of culture-based tests. For these reasons, molecular tests have been developed and claim to overcome most of these limitations. These molecular assays comprise multiplex-PCR-based assays (10, 11), isothermal amplification (LAMP)-based assays (12), and microarray-based assays (13, 14). Some of these assays have been evaluated using only a collection of well-characterized CPO but not with clinical samples and/or with a multisite approach.

In a previous study, we evaluated the sensitivity and specificity of a PCR-based method in a cartridge format (Xpert MDRO assay) for detecting carbapenemase genes in rectal and perirectal swabs run on the GeneXpert platform (Cepheid, Sunnyvale, CA). Testing was performed in parallel with a reference culture method (15). This molecular assay included targets for *bla*_KPC_, *bla*_NDM_, and *bla*_VIM_ genes but not for *bla*_OXA-48_ and *bla*_IMP-1_. In the current study, an updated version of the Xpert Carba-R (Cepheid, Sunnyvale, CA) was evaluated, which included all five gene targets. The evaluation was performed in a multisite prospective study for European marketing authorization under the European directive on *in vitro* diagnostic medical devices (16).

## MATERIALS AND METHODS

### Study design, prospective, and contrived specimens.

The study was conducted between July 2013 and February 2014 at two institutions in the United States (David Geffen School of Medicine, University of California, Los Angeles, CA and Westchester Medical Center, Valhalla, NY) and two institutions in Europe (Central Manchester University Hospitals NHS Foundation Trust, Manchester, United Kingdom and Hospital Ramón y Cajal, Madrid, Spain). Specimens included prospectively recovered rectal swabs (*n* = 383) from patients who provided informed consent to participate in the study or whose routine care included screening for CPO following local infection control policies. The protocol was approved by each institution's Ethics Committee. A double swab set (Venturi Transystem; Copan, CA) and transport medium (liquid Stuart transport swab; Copan) were used to collect and transport rectal swab specimens from eligible subjects. One swab was used for reference culture, susceptibility testing, and sequencing, and another swab was used for the Xpert Carba-R. To diminish the potential bias of sampling differences, the two swabs were gently rolled against one another before starting the Cepheid Xpert Carba-R assay and culture procedure.

Due to the low prevalence of organisms containing some of the carbapenemase genes, the prospectively collected clinical rectal swab specimens were supplemented with an additional 250 contrived specimens. To prepare the contrived specimens, unique, well-characterized carbapenemase-producing (*n* = 108) and non-carbapenemase-producing isolates (*n* = 142) were spiked into the negative rectal swab matrix (Table 1). Fifty percent of the specimens were prepared at concentrations near the analytical limit of detection (LOD) of the Xpert Carba-R assay (ranging from 1.1 × 10^2^ to 1.2 × 10^3^ CFU/swab depending on the carbapenemase gene), and the remaining specimens covered clinically relevant analyte concentrations (1×, 3×, and 10× LOD) (17). Isolates were seeded onto mock rectal swabs that were prepared by dipping swabs into an individual stool matrix that tested negative for carbapenemase genes. Nonseeded swabs that were dipped into individual stool matrix were prepared as controls.

**TABLE 1:** Xpert Carba-R assay results by target for clinical and contrived specimens

Xpert Carba-R assay result	Clinical specimens (*n* = 383)	Contrived specimens (*n* = 250)	All specimens (*n* = 633)
Positive (single and/or combined targets)	42	107	149
IMP-1	0	25	25
VIM	2	24	26
NDM	2	23	25
KPC	13	19	32
OXA-48	20	15	35
VIM + OXA-48	4	0	4
NDM + KPC	1	0	1
IMP-1 + NDM	0	1	1
Negative	341	143	484

Results from the Xpert Carba-R assay for the clinical and contrived specimens were compared to those of the reference culture and sequencing results.

### Reference culture, susceptibility testing, and sequencing.

One of the prospectively collected rectal specimen swabs was placed in 10 ml of MacConkey broth (General Laboratory Products, Yorkville, IL, USA) containing a 10-μg meropenem disk (BD, Franklin Lakes, NJ, USA) and was vortexed. After 20 to 24 h of incubation at 35°C, MacConkey broths were sent from the collecting sites to a central reference laboratory (Clinical Microbiology Institute, OR, USA) for further processing. Upon receipt at the central reference laboratory, an aliquot of 100-μl was removed from the MacConkey broth and inoculated onto a MacConkey agar plate after which a 10-μg meropenem disk was placed in the center of the plate. Colonies on the MacConkey agar plate that grew within 27 mm of the meropenem disk after an overnight incubation at 35°C were further tested for the presence of a carbapenemase, as described by Lolans et al. (18) with some modifications. Meropenem was used instead of ertapenem to improve the specificities for Pseudomonas spp. and Acinetobacter spp., which are intrinsically resistant to ertapenem. Colonies were also tested by a standard disk diffusion method, and those classified as carbapenem-nonsusceptible (i.e., intermediate or resistant to ertapenem, imipenem, or meropenem using the Clinical and Laboratory Standards Institute [CLSI] interpretive criteria [19]) were subjected to bidirectional DNA sequence analyses for the identification of the *bla*_KPC_, *bla*_NDM_, *bla*_OXA-48_, *bla*_IMP-1_, and *bla*_VIM_ genes. The bidirectional DNA sequence analyses were performed at an independent laboratory (ACGT Inc., Wheeling, IL, USA) using primers that were different from those used in the Xpert Carba-R assay.

All contrived swab specimens were processed at the central reference laboratory in the same manner as the clinical rectal swab specimens. They were inoculated into MacConkey broth with a meropenem disk and subsequently subcultured to MacConkey agar. Isolated colonies were tested for susceptibility.

A positive reference culture result for both clinical and contrived specimens was defined as the isolation of a carbapenem-nonsusceptible organism that contained a *bla*_KPC_, *bla*_NDM_, *bla*_OXA-48_, *bla*_IMP-1_, and/or *bla*_VIM_ gene confirmed by DNA sequence analysis. A negative reference culture result was defined as either a reference culture that did not yield any carbapenem-nonsusceptible organisms or the isolation of a carbapenem-nonsusceptible organism that did not contain any of the target carbapenemase genes by DNA sequence analysis.

To minimize bias in specimen analyses, the laboratory personnel performing the reference culture and DNA sequencing were not aware of the Xpert Carba-R assay results.

### Xpert Carba-R assay.

Xpert Carba-R assay testing was performed using the second swab from the double swab set with the GeneXpert platform. Testing was performed at the laboratories in each of the 4 participating institutions according to the manufacturer's recommendations and as previously described (15) within 24 to 48 h from collection. The assay has a run time of ∼47 min in the instrument. Quality control for the Xpert Carba-R assays consisted of one positive and one negative control. The former was composed of an Escherichia coli isolate containing a plasmid that included DNA fragments of all five target gene sequences, and the latter was the same E. coli isolate with the cloning vector but without the cloned fragments. The controls (product M219) were produced by Maine Molecular Quality Controls, Inc. (http://www.mmqci.com/qc-m219.php). The two controls were run on each day that specimens were tested. Study specimens were not tested until correct results were obtained for the negative and positive controls.

All contrived swab specimens were tested with the Xpert Carba-R assay in the same manner as the clinical rectal swab specimens.

### Data analysis, discrepant results, and statistics.

The Xpert Carba-R assay results were compared with reference culture and DNA sequencing results. An Xpert Carba-R-positive result was considered when the Xpert Carba-R assay detected the presence of at least one carbapenemase gene. For each specimen, results for each of the five carbapenemase target genes included in the Xpert Carba-R assay were reported separately, and these were compared to results for the five carbapenemase target genes obtained from reference culture and DNA sequencing.

A discrepant result was defined as a result obtained with the Xpert Carba-R assay that did not correlate with the results of reference culture and DNA sequencing of the same specimen. Discrepant testing was performed for only those specimens that were positive for a target gene by the Xpert Carba-R assay but did not show growth of colonies by culture. DNA was extracted from the MacConkey broth (100 μl) using the Qiagen DNeasy blood and tissue kit using the protocol for Gram-negative bacteria and amplified by PCR using primers corresponding to the targets that were positive by the Xpert Carba-R assay results. If one of the five targets was identified, bidirectional DNA sequence analysis was performed. A positive result was considered a true positive for discrepant resolution by the reference method in the analysis.

Discrepant testing was not performed for specimens that were negative for all target genes by the Xpert Carba-R assay but showed growth of colonies on the MacConkey agar plate and from which one of the five target genes was identified through DNA sequence analysis.

Sensitivity, specificity, and positive (PPV) and negative (NPV) predictive values were also calculated for each target carbapenemase gene (20).

Statistical analysis was performed using 95% confidence intervals (CIs) calculated with the Clopper-Pearson/Fisher exact CI (21) using Minitab version 16 (Minitab, State College, PA). Values for the Kappa coefficient, which gives a measure of the percentage of agreement between the Xpert Carba-R assay and the reference method (after resolution of discrepant results) beyond that expected by chance, were also calculated (22).

## RESULTS

### Xpert Carba-R assay results with prospective rectal and contrived swab specimens.

A total of 633 samples (383 clinical rectal swabs and 250 contrived specimens) were included. Overall, 149/633 (23.5%) samples were positive by the Xpert Carba-R assay, and 484/633 (76.5%) were negative. The Xpert Carba-R assay amplified 155 positive targets (26 *bla*_IMP-1_, 30 *bla*_VIM_, 27 *bla*_NDM_, 33 *bla*_KPC_, 39 *bla*_OXA-48_). In 6 samples, multiple targets were detected (4 *bla*_VIM_-*bla*_OXA-48_, 1 *bla*_IMP-1_-*bla*_NDM_, 1 *bla*_NDM_-*bla*_KPC_). Xpert Carba-R results stratified by clinical and contrived specimen type are shown in Table 1. Data analyses included discrepant resolution results (see below). All positive results were obtained within 32 to 48 min.

### Comparison of the Xpert Carba-R assay with the reference method.

Results from the Xpert Carba-R assay by individual target compared to the reference method (culture plus sequencing) for all specimens are shown in Table 2. There were a total of 633 specimens, each with results for five individual targets for a total of 3,165 results. Specimens that were negative by both the Xpert Carba-R assay and the reference method (3,004 total) were stratified as follows: 606 for IMP-1, 601 for VIM, 606 for NDM, 599 for KPC, and 592 for OXA-48.

**TABLE 2:** Results from the Xpert Carba-R assay and the reference method (culture plus sequencing) by individual target for combined clinical and contrived specimens

Xpert Carba-R assay	Reference method (culture plus sequencing)						
	IMP-1	VIM	NDM	KPC	OXA-48	Negative	Total
IMP-1	26	0	0	0	0	0	26
VIM	0	29	0	0	0	1	30
NDM	0	0	26	0	0	1	27
KPC	0	0	0	29	0	4	33
OXA-48	0	0	0	0	38	1	39
Negative	1	2	0	1	2	3,004	3,010
Total	27	31	26	30	40	3,011	3,165

Table 3 shows the overall performance of Xpert Carba-R compared with that of the reference method. The results were defined as positive for the Xpert Carba-R assay if any of the carbapenemase targets were positive, and they were defined as negative for the Xpert Carba-R assay if all of the carbapenemase targets were negative. On the combined set of clinical and contrived specimens, sensitivity, specificity, PPV, and NPV were 96.6% (95% CI, 92.2% to 98.9%), 98.6% (95% CI, 97.1% to 99.4%), 95.3%, and 99.0%, respectively. The Kappa index for this comparison was 0.933 (0.900 to 0.967).

**TABLE 3:** Overall Xpert Carba-R performance versus that of the reference method (culture plus sequencing) for combined clinical and contrived specimens

Xpert Carba-R assay	Reference method (culture plus sequencing)		
	No. positive	No. negative	Total No.
Positive	142	7	149
Negative	6	478	484
Total	148	485	633

The performance of the Xpert Carba-R assay for different targets, including CI, is shown in Table 4. Results included in Tables 2 to 4 were also analyzed when discrepant results were resolved (see below).

**TABLE 4:** Summary of Xpert Carba-R performance for different carbapenemase targets versus that of the reference method (culture plus sequencing) for combined clinical and contrived specimens

Target gene	Sensitivity (% [95% CI])	Specificity (% [95% CI])	PPV (%)	NPV (%)
IMP-1	96.3 (81.0–99.9)	100 (99.4–100)	100	99.8
VIM	93.5 (78.6–99.2)	99.8 (99.1–100)	96.7	99.7
NDM	100 (86.8–100)	99.8 (99.1–100)	96.3	100
KPC	96.7 (82.8–99.9)	99.3 (98.3–99.8)	87.9	99.8
OXA-48	95.0 (83.1–99.4)	99.8 (99.1–100)	97.4	99.7

### Discrepant results and resolution.

Six specimens (4 prospective and 2 contrived specimens) were negative for all target genes by Xpert Carba-R assay but were positive by the reference method (culture and DNA sequencing) and were considered to be false negatives. Bidirectional DNA sequence analysis identified 1 *bla*_IMP-1_, 2 *bla*_VIM_, 1 *bla*_KPC_, and 2 *bla*_OXA-48_ genes (Table 2). In addition, 18 specimens (15 prospective and 3 contrived specimens) were positive for at least one target gene by the Xpert Carba-R assay and were negative by the reference method (Table 5). Eleven of the 18 specimens were considered to be true positives after discrepant analyses, which included direct PCR analysis and sequencing from the MacConkey broth. Of these 11 specimens, there were 5 out of the 6 specimens in which multiple carbapenemase targets were detected by Xpert Carba-R assay. The remaining 7 specimens were considered to be false positives, as no PCR amplification was obtained from testing the MacConkey broth (Table 5). Targets detected by Xpert Carba-R assay were 4 KPC and one each of NDM, VIM, and OXA-48. In all of these cases, positive results were fairly late (threshold cycles [*C_T_*s] ranged from 26.2 to 28.2 for KPCs and were >31 for the remaining carbapenemases).

**TABLE 5:** Discrepant testing results^*a*^

Sample	Reference method		Target(s) detected by Xpert Carba-R assay	Discrepant analysis	
	Culture	Sequencing of isolate		DNA extraction and sequencing of MacConkey broth	Outcome^*b*^
A	No isolate	NA^*c*^	IMP-1	IMP-1	TP
B	Acinetobacter baumannii	No bands^*d*^	KPC	KPC	TP
C	Klebsiella pneumoniae	OXA-48	OXA-48, VIM	OXA-48, VIM	TP
D	No isolate	NA	OXA-48	OXA-48	TP
E	Klebsiella pneumoniae	OXA-48	OXA-48, VIM	OXA-48, VIM	TP
F	No isolate	NA	VIM	VIM	TP
G	No isolate	NA	KPC	KPC	TP
H	No isolate	NA	OXA-48, VIM	OXA-48, VIM	TP
I	No isolate	NA	OXA-48	OXA-48	TP
J	Enterobacter cloacae	KPC	KPC, NDM	KPC, NDM	TP
K	Klebsiella pneumoniae	OXA-48	OXA-48, VIM	OXA-48, VIM	TP
L	No isolate	NA	KPC	No target gene	FP
M	No isolate	NA	NDM	No target gene	FP
N	No isolate	NA	VIM	No target gene	FP
O	No isolate	NA	KPC	No target gene	FP
P	No isolate	NA	KPC	No target gene	FP
Q	No isolate	NA	KPC	No target gene	FP
R	No isolate	NA	OXA-48	No target gene	FP

a True-positive and false-positive results are shown.

b TP, true positive; FP, false positive.

c NA, not applicable.

d No bands indicate that the sample did not meet PCR product band size inclusion criteria.

## DISCUSSION

Detection of fecal carriers of CPO has become a routine clinical practice in many parts of the world and is recommended by public health organizations for the containment of the spread of these isolates (3, 23). Different approaches are used in different countries and institutions, with some culturing rectal swabs on chromogenic media. More recently, molecular approaches have been developed to increase detection sensitivity and decrease reporting time (7, 24). We evaluated, through a prospective multisite study, the Cepheid Xpert Carba-R assay, a PCR-based method in a cartridge format developed for detecting carbapenemase genes in rectal swabs and run on the GeneXpert platform. This assay was previously evaluated, but only three carbapenemase targets (*bla*_KPC_, *bla*_NDM_, and *bla*_VIM_) were included (15). In the current version, two additional targets were incorporated, *bla*_OXA-48_ and *bla*_IMP-1_, to broaden the scope of the carbapenemases detected. OXA-48 is increasingly recognized in most European countries, predominating in most of the Mediterranean countries, whereas IMP-1 is increasingly detected in Asia and Latin American countries (3, 25).

Overall, 633 specimens, including clinical rectal swabs and contrived samples, were studied, yielding the potential detection of 3,165 carbapenemase targets. Due to the absence or low prevalence of certain carbapenemase genes in organisms found in clinical specimens, such as IMP-1, VIM, or NDM carbapenemases, that reflects the local epidemiology of participating centers, contrived specimens were also included in the evaluation. This approach is normally used in molecular diagnostic platforms for *in vitro* and clinical evaluations.

For our evaluation, the LODs of different carbapenemase targets were previously calculated (see Materials and Methods) (17). Additionally, analytical reactivity (inclusivity) and potential cross-reactivity (exclusivity) were also calculated, yielding 100% detection of target-containing organisms and no cross-reactions with other resistance genes, respectively (17). Moreover, it is notable that, in our evaluation, we used a highly sensitive method to resolve potential discrepancies between the reference method (culture plus DNA sequencing) and the Cepheid Xpert Carba-R assay. This consisted of DNA extraction and sequencing from the selective enrichment broth (MacConkey with a 10-μg meropenem disk) inoculated with the original specimens. With this approach, overall performance was excellent with sensitivity, specificity, PPV, and NPV all higher than 95%. When performance was evaluated separately for each carbapenemase, the performance was best for IMP-1, and only sensitivities and positive predictive values for VIM and KPC targets were lower than 95%. Unlike other systems for direct detection of carbapenemase genes in rectal swabs, we did not see high false-positive rates for metallo-beta-lactamase targets (26).

Our multisite prospective evaluation was performed for the initial European marketing authorization of the Xpert Carba-R assay and was carried out under the European directive on *in vitro* diagnostic medical devices (16). A limitation of our evaluation was that the Xpert Carba-R assay included targets for widespread carbapenemases at the time of the evaluation but did not include some of the new emerging OXA-48 variants, such as OXA-181 and OXA-232 carbapenemases. OXA-181 is one of the most prevalent carbapenemases in South Africa and is increasingly recognized in Asia and Europe (27–29), while OXA-232 is also increasingly detected in Asia (30). Recent reports demonstrated that the Xpert Carba-R version evaluated in our study was unable to detect these two variants (31–33). Nevertheless, this was corrected in a novel Xpert Carba-R version recently marketed, which accurately detected these OXA-48 variants (34). The absence of detection of the OXA-181 carbapenemase was also noted with other commercial molecular assays that did not originally include this target. However, as in the Xpert Carba-R assay, subsequent modification of the assay allowed detection of OXA-181 (34).

In our study, with the exception of 6 specimens (Table 2) in which false-negative results were obtained, the Xpert Carba-R assay accurately detected all different targeted carbapenemase genes, including those encoding the KPC, NDM, VIM, IMP-1, and OXA-48 enzymes. The absence of detection in these 6 specimens was not associated with a specific carbapenemase and may be associated with low bacterial load in the rectal swab specimens.

An additional advantage of the Cepheid Xpert Carba-R assay is the ability to detect multiple resistance genes, an outcome not previously reported in other evaluations (31). This occured in 6 cases (Tables 1 and 5). In 4 of them, the reference culture method failed to detect this situation, but the genes were subsequently detected by PCR and DNA sequencing from the MacConkey broth. A potential explanation for these results may be that the organisms recovered by reference culture are present in larger amounts, which would limit the growth of the other organisms present in a lower proportion, or because both carbapenemase-bearing isolates were morphologically identical and one of them was likely missed during the selection of the colonies.

However, a positive result with the Xpert Carba-R assay but negative by culture may happen if the patient had been receiving antibiotics, or the organism may carry a modified sequence of the target gene, which was not expressed or was expressed at low levels.

In conclusion, the Cepheid Xpert Carba-R assay is an accurate technique for the detection of CPO in rectal swab specimens. This assay allows rapid identification of patients colonized with CPO, which can guide infection control programs designed to limit the spread of these organisms in health care settings.
